# Design of combination therapy for engineered bacterial therapeutics in non-small cell lung cancer

**DOI:** 10.1038/s41598-022-26105-1

**Published:** 2022-12-13

**Authors:** Dhruba Deb, Yangfan Wu, Courtney Coker, Tetsuhiro Harimoto, Ruoqi Huang, Tal Danino

**Affiliations:** 1 Department of Biomedical Engineering, Columbia University, New York, NY 10027 USA; 2 Herbert Irving Comprehensive Cancer Center, Columbia University, New York, NY 10027 USA; 3 Data Science Institute, Columbia University, New York, NY 10027 USA

**Keywords:** Non-small-cell lung cancer, Applied microbiology, Bacteria, Bacterial host response, Bacterial toxins, Cancer models, Cancer therapy, Biomedical engineering

## Abstract

Synthetic biology enables the engineering of bacteria to safely deliver potent payloads to tumors for effective anti-cancer therapies. However, a central challenge for translation is determining ideal bacterial therapy candidates for specific cancers and integrating them with other drug treatment strategies to maximize efficacy. To address this, we designed a screening and evaluation pipeline for characterization of bacterial therapies in lung cancer models. We screened 10 engineered bacterial toxins across 6 non-small cell lung cancer patient-derived cell lines and identified theta toxin as a promising therapeutic candidate. Using a bacteria-spheroid co-culture system (BSCC), analysis of differentially expressed transcripts and gene set enrichment revealed significant changes in at least 10 signaling pathways with bacteria-producing theta toxin. We assessed combinatorial treatment of small molecule pharmaceutical inhibitors targeting 5 signaling molecules and of 2 chemotherapy drugs along with bacterially-produced theta toxin and showed improved dose-dependent response. This combination strategy was further tested and confirmed, with AKT signaling as an example, in a mouse model of lung cancer. In summary, we developed a pipeline to rapidly characterize bacterial therapies and integrate them with current targeted therapies for lung cancer.

## Introduction

**Figure 1:**
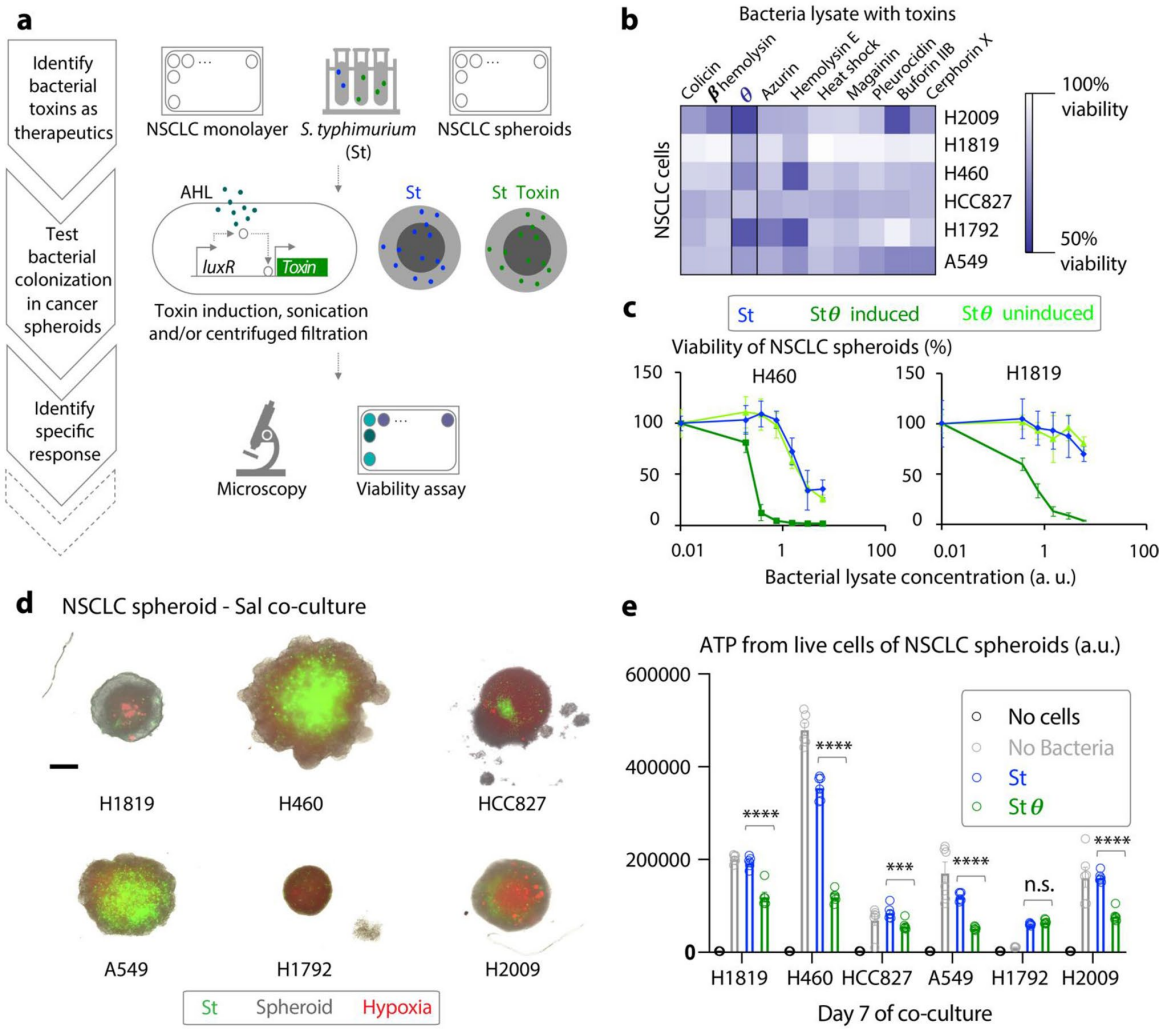
Identifying effective bacterial toxins for non-small cell lung cancer (NSCLC) therapy. (**a**) Schematic of the pipeline to identify bacterial toxins with potential as cancer therapeutics involving response of non-small cell lung cancer (NSCLC) to bacterial toxins in vitro, colonization of bacteria in NSCLC spheroids. (**b**) 2D monolayer screen using MTT viability assay to study the response of 6 NSCLC lines to 10 engineered bacterially secreted toxins. Fresh lysates of engineered *S. typhimurium* EHL1301 were prepared and were normalized for optical density before adding to the NSCLC cultures grown in 96-well flat bottom plates. The heatmap represents the median of percent viability (n = 8). (**c**) Viability of 2 NSCLC spheroids under 3 treatment conditions—serially concentrated lysate of *S. typhimurium* producing: (1) GFP and θ toxin (in presence of AHL) abbr. “Stθ induced”, (2) GFP and low amount of θ toxin due to leaky LuxR promoter (in absence of AHL) abbr. “Stθ uninduced”, and, (3) GFP only (n = 4) abbr. “St”, assessed by Cell Titer Glo 3D. Error bars represent standard deviation (n = 4). (**d**) Representative fluorescence microscopy images of 4-day old co-culture of 6 NSCLC spheroids (grey) with *S. typhimurium* producing GFP (green) and stained with hypoxia dye (red). Scale bar = 200 μM. (**e**) Viability of NSCLC-*Salmonella* co-culture spheroids using Cell Titer Glo 3D assay at day 7 (n = 6). Live *S. typhimurium* expressing θ toxin (Stθ) upon induction with AHL is abbreviated as “Stθ.” Significant change (**** = p < 0.0001, *** = p < 0.001, ** = p < 0.01, * = p < 0.5, n.s. = not significant) was determined by paired, two-tail *t* test, and error bars represent standard deviation.

Bacteria have been investigated for cancer therapy for many centuries, as exemplified by William Coley’s injections of *S. pyogenes* and *S. marcescens* into patients with inoperable bone and soft tissue sarcomas that induced remissions^1,2^. Since then, attenuated strains of bacteria such as *M. bovis, Bifidobacterium spp., S. typhimurium, L. monocytogenes, and C. novyi,* have been engineered to deliver therapeutics for solid tumors of the bladder, liver, colon, breast and brain in clinical trials (NCT02015104, NCT03358511, NCT04589234, NCT01967758, NCT01924689). There has been little attention paid to primary lung cancers yet there are several reports of an existing lung tumor microbiome^3–6^ presenting a natural case for bacterial therapies. Lung cancer is responsible for the highest number of cancer-related deaths worldwide and is also one of the most heterogenous type of cancer^7–9^. This heterogeneity of lung cancer along with the large search space of parameters for engineering bacterial strains, genetic circuits, and therapeutic payloads has produced a challenge in narrowing down a specific bacterial therapeutic for a particular type of lung cancer. Here, we focus on non-small cell lung cancer (NSCLC), the most common of lung cancer cases^10^. We establish an experimental pipeline to identify and characterize (Fig. 1a) therapeutics delivered by *Salmonella typhimurium* to match with NSCLC models and combine their benefit with current targeted therapies.

## Results

To identify bacterial toxins as effective therapeutics in lung cancer, we initially tested the viability of a subset of genetically diverse NSCLC cell lines upon exposure to an array of bacterial toxins. To identify a robust therapeutic, we chose a diverse array of NSCLC lines (ATCC): H460 has overexpression of TP53 mRNA, A549 has mutated KRAS, and HCC827 has mutated EGFR. H1819 and H2009 were derived from patients after administration of chemotherapy and radiation. H460 was derived from pleural fluid while H1792 was derived from a metastatic site. The bacterial toxins were previously engineered to be produced under inducible circuits of *S. typhimurium* EHL1301^11,12^. We induced therapeutic production in *S. typhymurium* and applied bacterial lysates to cells, which identified θ toxin as having the highest effect on viability among all bacterial toxins tested (Fig. 1b, Supplementary Fig. 1). θ toxin is a pore forming toxin from *C. perfringens*^13^ and was previously shown to have efficacy against murine colon cancer cells^11^. To model more physiologic conditions that occur within tumors containing poorly vascularized hypoxic regions where therapeutic efficacy is thought to be reduced^14^, we tested the effect of θ toxin on spheroids derived from a subset of our cell lines. We found that lysate of *S. typhymurium* expressing θ toxin (**Stθ**) induced significantly higher cell death (p < 0.001) within the spheroids in a dose-dependent manner, as compared to lysate of non-induced *S. typhymurium* or lysate of *S. typhymurium* alone (Fig. 1c). Furthermore, these spheroids responded to purified θ toxin as well (ATCC, BTX-100) above 0.5 μg/mL concentration (Supplementary Fig. 2). Taken together, we identified θ toxin as a promising therapeutic to be delivered by bacteria for several NSCLCs.

To selectively deliver therapeutics to tumor cells, bacteria need to be able to efficiently colonize tumors in spite of the production of payloads. Previous studies have established that live *S. typhymurium* can colonize murine colon cancer spheroids due to tumor-specific signatures, such as hypoxia, acidic pH and lactate^15–17^. We first assayed if live *S. typhymurium* were capable of colonizing hypoxic regions within the NSCLC spheroids. We found that the GFP-labeled bacteria colonized near dye-labeled hypoxic regions within the spheroid core (Fig. 1d), where they persisted for the duration of one week (Supplementary Fig. 3). Next, we assayed the viability of NSCLC spheroids containing live *S. typhymurium* when θ toxin expression was induced with AHL. We found that live Stθ significantly reduced viability of the majority of NSCLC spheroids, as compared to colonized live *S. typhymurium* alone (Fig. 1e). An extended analysis demonstrated that reduced viability was achieved in all spheroids within 2 weeks of Stθ exposure (Supplementary Fig. 4). The ability of live Stθ to persistently colonize the spheroid core, while robustly reducing viability of heterogenous NSCLC cell types, encouraged the extension of this approach to in vivo conditions.

**Figure 2:**
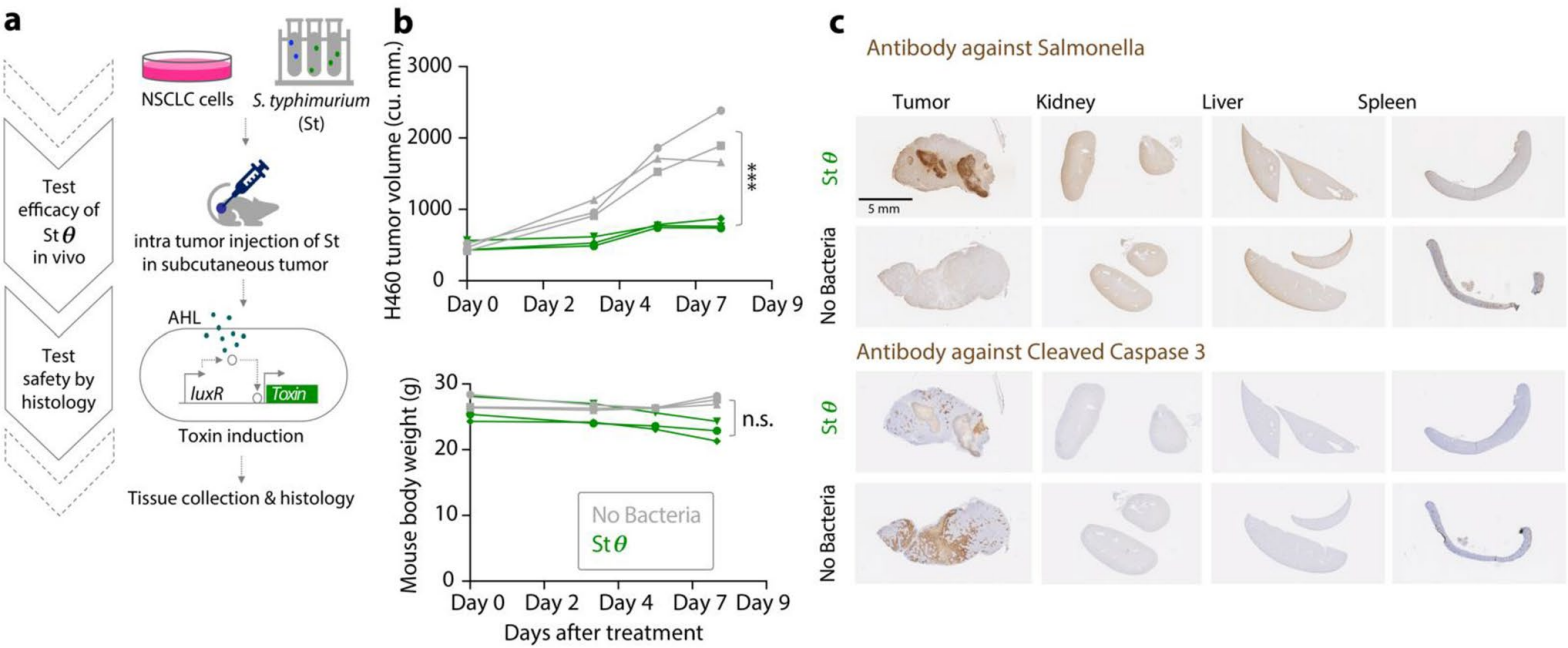
Testing the efficacy of bacterial toxins in NSCLC in vivo. (**a**) Schematic of the second part of the pipeline to test the efficacy and safety of bacterial toxins via intratumoral delivery (**b**) (top) Response of H460 subcutaneous tumors in NSG mice to intratumor delivery of live *S. typhimurium* secreting θ toxin upon induction with AHL injection. Treatment began once tumors reached ~ 500 cu.mm., using 40 uL of live bacteria in sterile 1 × DPBS (vehicle) with concentration 4.5 × 10^8^ CFU/mL (OD 1), twice a week, (bottom) body weight of mice including tumors’ weight, significant change (*** = p < 0.001, n.s. = not significant) was determined by two-way repeated Anova (n = 3). (**c**) Representative histology images of formalin-fixed, paraffin-embedded tumors and peripheral organs stained for *S. typhimurium* serotype ‘O’ specific antibody and cleaved Caspase 3 with or without bacteria injection.

We next explored the efficacy of Stθ in a mouse tumor model, which would locally deliver θ toxin in vivo while reducing systemic toxic effects. Specifically, we administered live bacteria intratumorally in tumor xenografts grown from a NSCLC cell line (H460) and induced the production of θ toxin 1 day after bacteria injection (Fig. 2a). This assay resulted in a 2.5-fold reduction in tumor growth within a week (Fig. 2b, top, p-value < 0.001, Supplementary Fig. 5). Importantly, tumor control was achieved without inducing systemic toxicity after administration of high concentration of Stθ (4.5 × 10^8^ CFU/mL and 40 μL per tumor), as assessed by weight change (Fig. 2b, bottom), and the absence of detectable *S. typhymurium* via IHC assay from peripheral organs at the endpoint (Fig. 2c, top). In addition, none of the peripheral organs showed signs of apoptosis, as indicated by the lack of cleaved caspase 3 signal, compared to the tumors (Fig. 2c, bottom, Supplementary Fig. 6). As immortalized Human Bronchial Epithelial Cells (HBECs) showed moderate level of response to the toxins (Supplementary Fig. 7), we routinely examined behavioral points of the mice such as activity, aggression, bite reflex, posture, presence of straub tail, seizures, and overall signs of morbidity upon Stθ exposure. Our experiments demonstrated lack of these behavioral changes. In summary, our studies point to the safety of this approach, reducing concerns regarding potential systemic toxicity associated with intratumoral use of live *S. typhymurium *in vivo.

**Figure 3:**
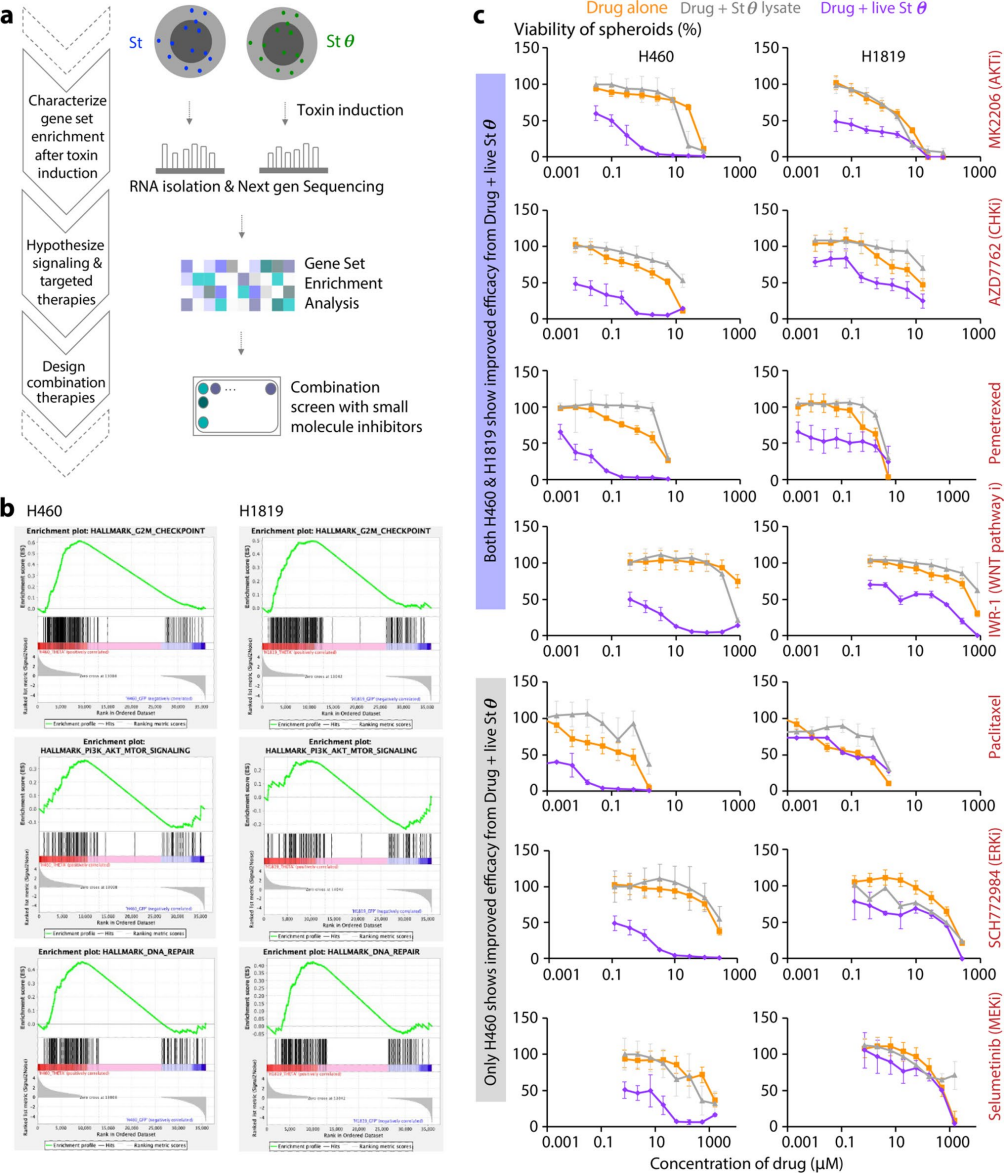
Characterizing NSCLC’s response to bacterial therapeutics and designing combination therapies. (**a**) Schematic of the third part of the pipeline to characterize the NSCLC’s response to *S. typhimurium* -secreted theta toxin, using RNA sequencing, bioinformatic analysis, designing and testing combination treatment regime in vitro. (**b**) Gene Set Enrichment Analysis (GSEA) plots (representatives of 3 gene sets) from analyzing Next Gen RNA-sequencing data of 2 NSCLC spheroids, H460 and H1819 co-cultured with live *S. typhimurium* expressing θ toxin (Stθ) and GFP compared to spheroids with live *Salmonella* producing only GFP (n = 3). (**c**) Viability of 2 NSCLC spheroids, H460 and H1819, treated with 7 small molecule inhibitors (MK2206, AZD7762, Pemetrexed, IWR-1, Paclitaxel, SCH772984 and Selumetinib, targeting specific signaling identified by GSEA, under 3 treatment conditions: (1) Drug alone, (2) Drug and bacterially secreted θ (Stθ) in co-culture, (3) Drug and lysates of *S. typhimurium* producing θ toxin (n = 3, error bars represent standard deviation). Drug concentrations are in μM unit.

**Figure 4:**
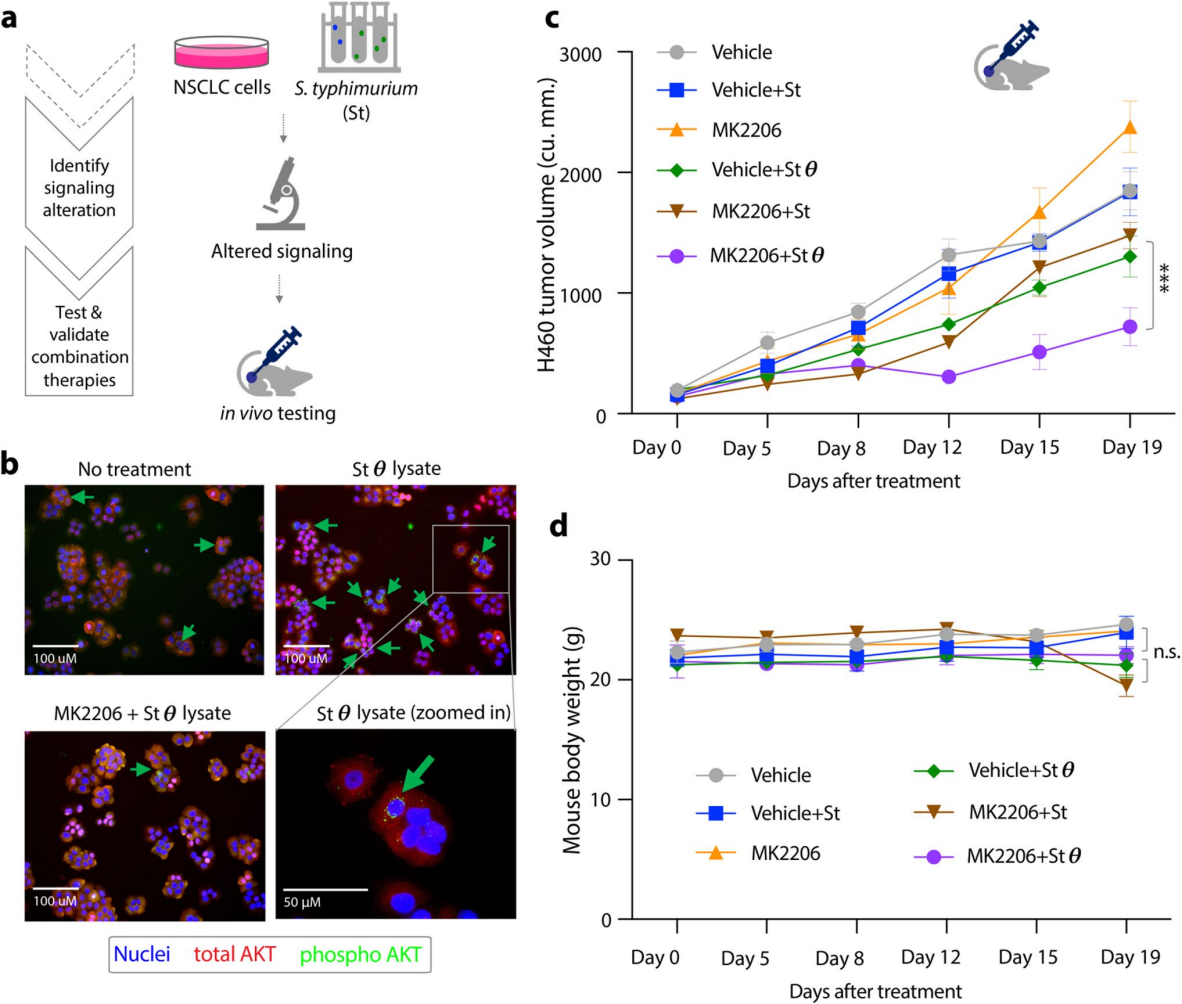
Combining AKT inhibitors and bacterial therapeutics for NSCLC therapy. (**a**) Schematic of the fourth part of the pipeline to validate the efficacy of one of the combination therapies. (**b**) AKT signaling in H460 cells upon θ toxin and MK2206 treatment: *S. typhimurium* was grown in presence of AHL to produce θ toxin and were lysed and filtered. H460 cells in monolayer were treated with θ toxin filtrate for 30 min, with or without 10 mM MK2206. Indirect immuno-fluorescence was carried out with antibodies against total AKT (red), phospho-AKT (Ser473) (green). Nuclei were stained with DAPI (blue). (**c**) Response of H460 subcutaneous tumors in NSG mice to 6 treatment cohorts (n = 3 or 4 per cohort): Vehicle, Vehicle + *S. typhimurium* producing GFP abbr. “St”, MK2206 targeting AKT, Vehicle + *S. typhimurium* producing both GFP and θ toxin abbr. “Stθ”, MK2206 + St, and, MK2206 + Stθ. Treatment began once tumors reached ~ 150 cu.mm. twice a week. Live Salmonella was injected intratumorally suspended in 40 μL of sterile 1 × DPBS (vehicle) with concentration of 4.5 X 10^7^ CFU/mL followed by subcutaneous injection of AHL, a day after, to induce θ toxin secretion within the tumor. MK2206 drug was delivered by oral gavage as 240 mg/kg dose with 30% Captisol in 1 × DPBS as vehicle. Significant change in tumor size (*** = p < 0.001) was determined by two-way repeated ANOVA and error bars represent standard error. (**d**) Body weight of mice measured throughout the treatment schedule, error bars represent standard error (n = 3 or 4), n.s. = not significant.

Can we improve Stθ by combining with current standard of care chemotherapies as well as small molecule inhibitors being tested in clinical trials? To narrow down potential drugs to combine with Stθ treatment, we aimed to gain mechanistic insight into cellular pathways altered in two of the Stθ-responder spheroids (H460 and H1819, Fig. 3a). We compared genome-wide transcriptional profiling (RNA-seq) of tumor cells derived from spheroids housing live *S. typhymurium* in their hypoxic cores in presence or absence of producing θ toxin. Gene Set Enrichment Analysis (GSEA) of Next-Gen sequencing revealed significant changes in at least 10 signaling pathways shared by both NSCLC cell lines upon Stθ treatment (Fig. 3b and Supplementary Fig. 8, normalized p-value < 0.5 and falls discovery rate q-value < 0.5). Specifically, cell cycle checkpoint, PI3K/AKT/mTOR signaling and DNA repair pathways were among the most significantly enriched gene sets in both H460 and H1819. As top 50 differentially expressed genes from H1819 and H460 did not show modest overlap (Supplementary Fig. 9), we selected 7 small molecule inhibitors specific to lung cancer therapeutic landscape that are well known (NCT01294306, NCT00744900, NCT03392246)^18,19^ to target the significantly enriched pathways in our analysis. We hypothesized—if under Stθ treatment, H1819 and H460 are dependent on the signaling through these pathways for their survival, a combinatorial strategy with each drug and Stθ would eliminate more cancer cells than the single treatments. Indeed, when combinatorically treated with each drug and Stθ, 4 out of 7 combinations showed robust efficacy across both spheroids (Fig. 3c) compared to drug-only or lysate of *Salmonella* expressing θ toxin. Notably, H1819 showed more resistance to 3 out of 7 combination therapy, and may represent more drug-resistant disease, as it was derived from a patient previously treated with both chemotherapy and radiation (ATCC). Interestingly, this benefit was not observed when the combinatorial treatment was tested on spheroids derived from mouse lung cancer cells with genetically modified TP53 and KRAS (Supplementary Fig. 10) suggesting this improved efficacy is specific to H1819 and H460 as indicated by their transcriptional profiling. Taking AKT signaling as an example, we observed high expression of phopho-AKT (Ser473) upon treatment with bacterial θ toxin and this signal was reduced in presence of MK2206 drug, (Fig. 4a,b) which is known to block AKT-phosphorylation at Ser473. This data suggests biological changes induced by the θ toxin treatment in NSCLC cells can be exploited to design new combination therapies. Specifically, NSCLC cells may upregulate AKT phosphorylation upon θ toxin treatment and blocking AKT phosphorylation with MK2206 achieves improved efficacy.

Lastly, to test the efficacy of this combination therapy in vivo, we treated tumor xenografts with Stθ and MK2206 drug. For effective comparison with MK2206 + Stθ treatment, we designed multiple control cohorts: vehicle only, MK2206 alone, Stθ alone, live *S. typhymurium* alone, and, MK2206 + live *S. typhymurium*. Additionally, to observe the benefit of combination therapy at lower dosage and earlier time points, we intratumorally dosed bacteria (4.5 × 10^7^ CFU/mL and 20 μL per tumor) and started treatment when tumors reached ~ 150 mm^3^. The cohort treated with MK2206 alone or with live bacteria producing no toxin showed no difference in tumor growth from the cohort treated with only vehicle (Fig. 4c). However, mice that received MK2206 + Stθ treatment showed significantly reduced tumor growth (p-value < 0.001) compared to other control cohorts. No significant reduction in total body weight was detected in any treatment groups (Fig. 4d). Taken together, Stθ showed improved efficacy and no additional toxicity when combined with MK2206 targeted therapy in vivo.

## Discussion

We established a strategy to identify combination treatments for genetically-distinct solid tumors by assessing their response to bacteria therapies. Due to the rapid advances in synthetic biology, the ability to create engineered bacterial therapies far outpaces the throughput of animal-based testing, thus creating a major bottleneck for clinical translation. Here, we utilized NSCLC spheroids as a model for solid tumors which are three-dimensional and more physiological relevant in predicting bacterial therapy responses in mouse cancer models^11,12,15^. The combination with RNA-seq based identification of small molecule inhibitors enabled rapid and parallel assays for selection of effective therapies, which were then validated for safety and efficacy in a mouse model.

A limitation of our current pipeline is the low number of animals per cohort in the in vivo studies. Increasing the number of animals and assessing the overall survival upon treatment will be important, specifically if this pipeline is utilized for understanding the mechanism of resistance to bacterial therapeutics. Nevertheless, our approach addresses an impediment to the development of new combination therapies for solid tumors – namely, the narrow therapeutic window before significant systemic toxicity is observed^20–22^. As the toxins by themselves are not selectively detrimental to the cancer cells, selective delivery of the toxin by the live bacteria in the tumor is crucial to avoid systemic toxicity. Several species of bacteria can selectively colonize solid tumors, primarily due to reduced immune surveillance in tumor cores, and can be controlled to deliver therapeutic payloads after colonizing tumors^23–25^. Furthermore, attenuations of bacteria, such as those previously made to *S. typhimurium* reduce pathogenicity and immunogenicity, which have led to clinical trials where safety has been demonstrated^26,27^. Thus, our treatment strategy has the potential to increase therapeutic windows for NSCLC by combining the benefits of both bacteria therapies and previously developed safe pharmacological inhibitors.

Although this study focused on tumor cells in response to θ toxin treatment, going forward it can be similarly used to determine the response of immune cells to Stθ treatment to establish a comprehensive safety profile. Additionally, while we focused on NSCLC, our strategy can be expanded to other lung cancers and to the cancer of other organs. We envision fast and selective expansion of this pipeline to improve treatment efficacy and safety for solid tumors.

## Methods

### Monolayer and spheroid culture of animal cells

NSCLC cells, H1819, H460, A549 (ATCC cat. no. CRL-5897, HTB-177 and CCl-185), H2009, H1792, HCC827 (Dr. John D. Minna, University of Texas Southwestern Medical Center) were cultured in growth media containing RPMI 1640 (Gibco Thermo-Fisher cat.no.11835030) with 5% Fetal Bovine Serum (FBS) (Gibco Thermo-Fisher cat.no.10-437-028), 1% Penicillin – Streptomycin (CellGro Thermo-Fisher cat.no. 15-070-063) inside 5% CO_2_ in 37 °C CO_2_ incubator (Thermo Scientific). HBECs (Dr. John D. Minna) and mouse lung cancer cells with genetically modified TP53 and KRAS (Dr. Tyler Jacks) were cultured in Keratinocyte-SFM and supplements (Thermo-Fisher cat.no. 17005042) and DMEM (Gibco Thermo-Fisher cat.no. 11320033) with 10% FBS, respectively. Subcultures were prepared followed by trypsinization with 0.05% Trypsin EDTA (Gibco Thermo-Fisher cat.no. 25-300-054), neutralization by the growth media for human NSCLCs, mouse cells, and by Trypsin Neutralizer Solution (Thermo-Fisher cat.no. R002100) for HBECs, and counted by Trypan blue (Gibco Thermo-Fisher cat.no. 15250061) staining using counter slides (Thermo-Fisher cat.no. C10228) in Cell counter (Invitrogen Thermo-Fisher cat.no. AMQAX2000). Spheroids were cultured by seeding 2,500 cells in 100 μL of growth media per well in 96-well, clear, round bottom, ultra-low attachment plates (Corning Costar Sigma cat.no. CLS7007-24EA)^11,12^. All cells were tested for sterility and authenticity by IDEXX laboratories (Maine, USA).

### *S. typhimurium* culture and lysate preparation

Attenuated strain of *S. typhimurium*: ELH1301 (Dr. Elizabeth Hohmann) was cultured in LB agar (Lennox Thermo-Fisher cat.no. 22700025) growth medium inside 37 °C incubator shaker (Thermo-Fisher, SHK6000). Optical density was measured by NanoDrop One^c^ 368 (Thermo-Fisher, ND-ONEC-W). Utilizing plasmids—Constitutive GFP (pTH01) and AHL-inducible therapeutics (pTH05), *S. typhimurium* was engineered to secrete 10 bacterial toxins^11,12^ upon AHL induction (N-(β-Ketocaproyl)-L-homoserine lactone, Sigma, K3007). All bacteria were grown with appropriate antibiotics selection—100 μg ml^−1^ ampicillin (Sigma-Aldrich cat.no. A5354) and/or, 50 μg ml^−1^ kanamycin (Sigma-Aldrich cat.no. 10106801001). Toxin production was induced by adding 10 nM N-(β-Ketocaproyl)-L-homoserine lactone (AHL) (Sigma Aldrich cat.no. O1764). When the culture reached optical density value of 1, the culture was sonicated (Fisher cat.no. FB505) and filtered (0.22 micron, Millipore-Sigma Steriflip™ cat.no. SE1M179M6). No phosphatase cocktail was added for this study. Concentrated lysates were prepared by passing them through ultracentrifuge filters (Millipore-Sigma, Amicon cat.no. ACS505024) matching with the size of the θ toxin^13^.

### In vitro treatment, MTT and Cell Titer Glo 3D viability assays

For monolayer viability assay, animal cells were allowed to adhere to the wells of Nunc 96-Well flat bottom plates (Thermo-Fisher cat.no. 167008) for a day before adding bacterial lysates along with 25 μg/ml gentamicin (Thermo-Fisher cat.no. 15-750-060). After 4 days, the viability was assessed by MTT assay (Sigma cat.no. TOX1-1KT) by measuring the colorimetric output in a TECAN Infinite M200 Pro plate reader. For spheroid viability assay the spheroids were cultured in 96-well, round bottom plates in growth media until they formed central hypoxic cores^11,12^ before adding bacterial lysates with 25 μg/ml gentamicin. Small molecule inhibitors: AZD7762, Selumetinib, SCH772984, Paclitaxel, Pemetrexed, MK2206, IWR-1 (Med Chem Express cat.no. HY-10992, HY-50706, HY-50846, HY-B0015, HY-10820, HY-10358, HY-12238, respectively) were diluted in DMSO (Millipore Sigma cat.no. D2650) to prepare stock solutions. After 4 days of treatment, the viability was assessed by Cell titer Glo 3D assay (Promega Thermo-Fisher cat.no. G9683) by measuring the luminescence in the plate reader.

### NSCLC spheroid and Salmonella co-culture, staining, microscopy and image analysis

Overnight culture of Salmonella was incubated with 3–4 days old NSCLC spheroids. The co-culture incubation time and gentamicin concentration after washing away the bacteria in the media was optimized based on the type of the spheroids to achieve moderate level of bacteria inside the spheroids^11,12^. Therapeutic expression was induced by adding AHL-containing growth media after 2–3 days and continued to be refreshed every 3 days. Hypoxia staining was carried out using Image-iT™ Red Hypoxia Reagent (Thermo-Fisher cat.no. H10498). Indirect immunofluorescence was carried out with fixing and permeabilization using 4% paraformaldehyde (Electron Microscopy Sciences EM grade Thermo-Fisher cat.no. 50-980-487) for 20 min in room temperature, washing with 0.1% Tween-20 (Thermo-Fisher cat.no. AAJ20605AP) in 1X DPBS (Thermo-Fisher cat.no. 14-190-250) 3 times each for 5 min, blocking using 5% Bovine Serum Albumin (BSA Thermo-Fisher cat.no. BP1600100) in washing buffer for 1 h in room temperature, primary antibodies—pan AKT mouse mAb (Cell Signaling cat.no. 2920) in 1:200 dilution, phospho-AKT (Ser473) rabbit mAb (Cell Signaling cat.no. 4060) in 1:100 dilution in blocking buffer, incubated at 4 °C overnight, secondary antibodies—Alexa Fluor® 647 anti-mouse (Thermo-Fisher cat.no. 50-164-553), Alexa Fluor® 488 anti-rabbit (Thermo-Fisher cat.no. A-11008) 1:1000 in blocking buffer, and, nuclear stain DAPI (Sigma-Aldrich cat.no. D9542) 1:4000 dilution in blocking buffer incubated at room temperature for 2 hrs^28^. Fixed-cell images and spheroid images were acquired using EVOS FL Auto 2 Cell Imaging Systems microscope and Celleste Imaging Analysis software (Thermo-Fisher). Live spheroid images were acquired using Nikon TiE microscope (equipped with Okolab stage top incubator, Andor Zyla sCMOS camera, and Lumencor Spectra-X Light) and Nikon Elements software. Analysis of spheroid sizes over time was carried out using FIJI (NIH)^29^.

### RNA isolation, sequencing and data analysis

RNA was isolated from H460 and H1819 spheroids (after co-cultured with *Salmonella-*secreted θ toxin) using RNA easy mini kit (Qiagen cat.no. 74134), quantified by NanoDrop One^c^ 368, and, the quality was assayed by Agilent bioanalyzer (Molecular Pathology Shared Resources, Columbia University). RNA-seq was carried out by Illumina NovaSeq 6000 and analyzed using DESeq2 (Bioconductor) and Kallisto (Patcher Lab) at Columbia Genome Center. Gene set enrichment analysis was carried out using GSEA software^30^.

### H460 subcutaneous tumor, treatment with live bacteria and small molecule inhibitor

All animal experiments were approved by the Institutional Animal Care and Use Committee (Columbia University, protocol AC-AABQ5551). 5 × 10^6^ H460 cells per mouse in 400 μL of 0.9% saline were injected subcutaneously in the right hind flank of 4–6 weeks old female NSG mice. Tumor volume was quantified using calipers to measure the length, width, and height of each tumor (V = ½ × L × W^2^). When tumors reached an average size of 400mm^3^, mice were randomized and assigned to the different treatment groups. *S. typhimurium* was prepared from overnight culture, controlled for OD 0.1 and concentration of 4.5 × 10^7^ 0r 4.5 × 10^8^ CFU/mL CFU/mL and intratumorally injected at a concentration of 5 × 10^8^ cells per mL in 1X PBS with total volume of 20–40 μL per tumor^11,12^. 0.5 mL of 10 μM AHL was injected subcutaneously the day after bacterial treatment to induce therapeutic expression. MK2206 was dissolved in 30% captisol (Selleck Chemicals cat.no. S4592) in 1X PBS and injected in 240 mg/kg concentration by oral gavage, twice a week. Animal experiments were carried out independently at Danino lab and at The Oncology Precision Therapeutics and Imaging Core (OPTIC) in Columbia University. We confirm that all methods were performed in accordance with the relevant guidelines and regulations.

### Tissue collection, processing, staining and imaging

Mice were euthanized per IACUC protocol. Immediately after euthanasia, tumors, kidneys, spleen, and liver were collected and fixed using 10% neutral buffered formalin (Ajax FineChem™ Thermo-Fisher cat.no. AJA2518-5), for 72 h, at which point they were transferred into 70% Ethanol (EtOH) and stored at 4 °C. Tissues were sectioned and transferred to cassettes. Further processing, sectioning, staining for H&E, immunohistochemistry using Serotype “O” primary antibody (Thermo-Fisher cat.no. PA1-7213) and caspase 3 (cleaved Asp353, Invitrogen, Thermo-Fisher cat.no. PA5-105271) were carried out at the Histology and Imaging Core, University of Washington. All slides were digitally scanned using Nanozoomer (Hamamatsu).

### Ethics declarations

The authors declare no competing interests. All animal experiments were approved by the Institutional Animal Care and Use Committee (Columbia University, protocol AC-AABQ5551). All methods are reported in accordance with ARRIVE guidelines (https://arriveguidelines.org) for the reporting of animal experiments. For tumor-bearing animals, euthanasia was required when the tumor burden reached 2 cm in diameter or after recommendation by the veterinary staff. Euthanasia was carried out in a CO_2_ chamber with a regulator and flow meter to maintain a fill rate of 30–70% of chamber volume per minute for 5 min. Gas flow was maintained for at least a 1 min after cessation of respiration. A secondary physical method of cervical dislocation is followed.

## Supplementary Information

Supplementary Figures.

## Data Availability

The high-throughput RNA sequencing expression profiles generated in this study were deposited at the NCBI Gene Expression Omnibus Database (https://www.ncbi.nlm.nih.gov/geo/query/acc.cgi) with accession number—GSE210560 and reviewer token—kpinykqqbhcpfwr. A subset of microscopy images are also available upon request.
