# Supplementary material for: Design of combination therapy for engineered bacterial therapeutics in non-small cell lung cancer
Source: Sci Rep. 2022 Dec 13;12:21551. doi: 10.1038/s41598-022-26105-1 (PMC9748036; doi:10.1038/s41598-022-26105-1)
Supplement: Supplementary file 1 — Supplementary Figures. [file 41598_2022_26105_MOESM1_ESM.pdf]

Supplementary information

Design of combination therapy for engineered bacterial therapeutics in non-small cell lung cancer

Dhruba Deb[1], Yangfan Wu[1], Courtney Coker[1], Tetsuhiro Harimoto[1], Ruoqi Huang[1], Tal Danino[1,2,3]

[1]Department of Biomedical Engineering, [2]Herbert Irving Comprehensive Cancer Center, [3]Data Science Institute, Columbia University, New York, NY 10027, USA. Electronic address: td2506@columbia.edu

Supplementary Figures

Suppl. Fig. 1

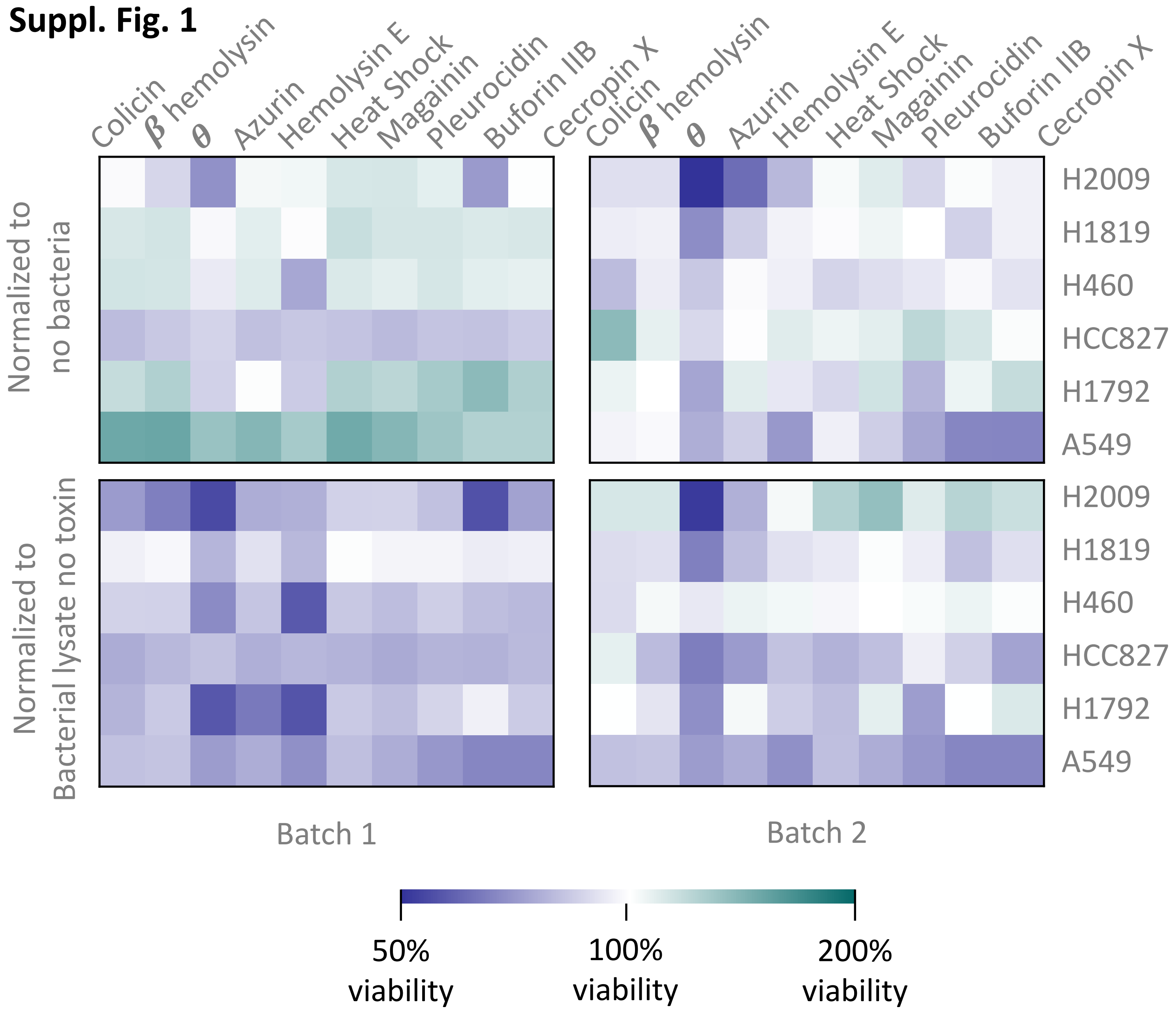

Suppl. Fig. 1: Response of NSCLC lines to bacterially secreted toxins to monolayer assay in batch replicates
2D monolayer screen using MTT viability assay to study the response of 6 NSCLC lines to 10 previously engineered bacterially secreted toxins. For each batch, fresh lysates of engineered *S. typhimurium* EHL1301 were prepared and were normalized for optical density before adding to the NSCLC monolayer cultures grown in 96-well flat bottom plates. The heatmap represents the median of percent viability (n=8 for plate replicates). Top row represents data normalized to no bacteria control. Bottom row represents data normalized to bacterial lysate without engineered toxins.

Suppl. Fig. 2

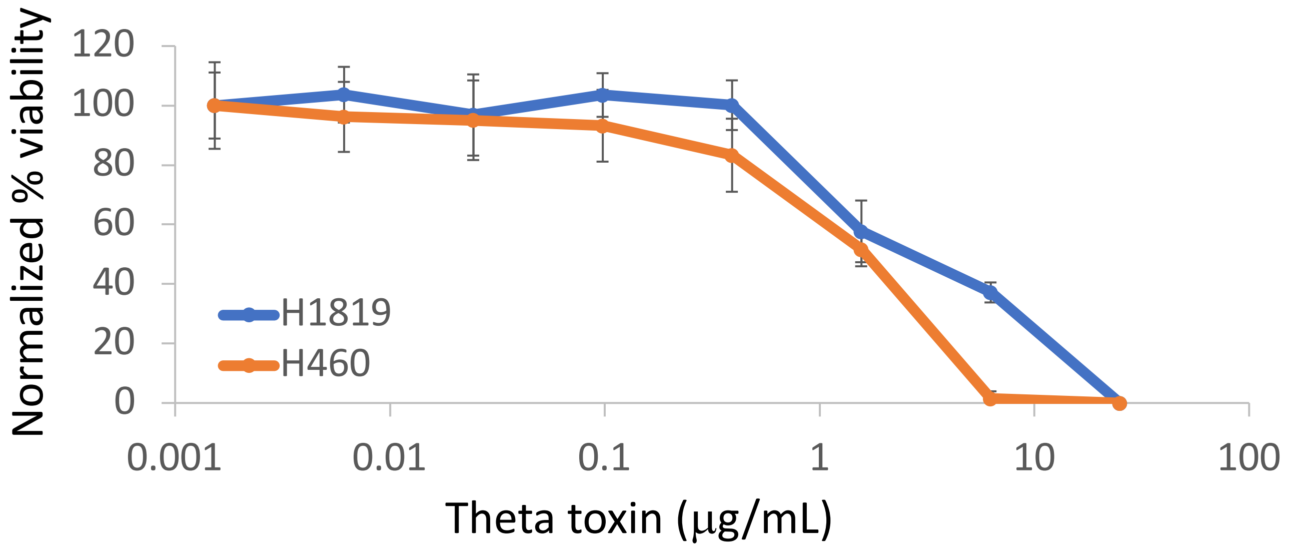

Suppl. Fig. 2: Response of NSCLC spheroids to purified theta toxin (ATCC)
Response of 2 NSLC spheroids to purified Theta toxin purchased from ATCC assessed by Cell Titer Glo 3D. Error bars represent standard deviation (n=3). Toxin concentration is in μg/mL unit. The toxin was dissolved in spheroid growth media and added after the spheroids developed hypoxic cores. Luminescence was measured after 96 hours of treatment.

Suppl. Fig. 3

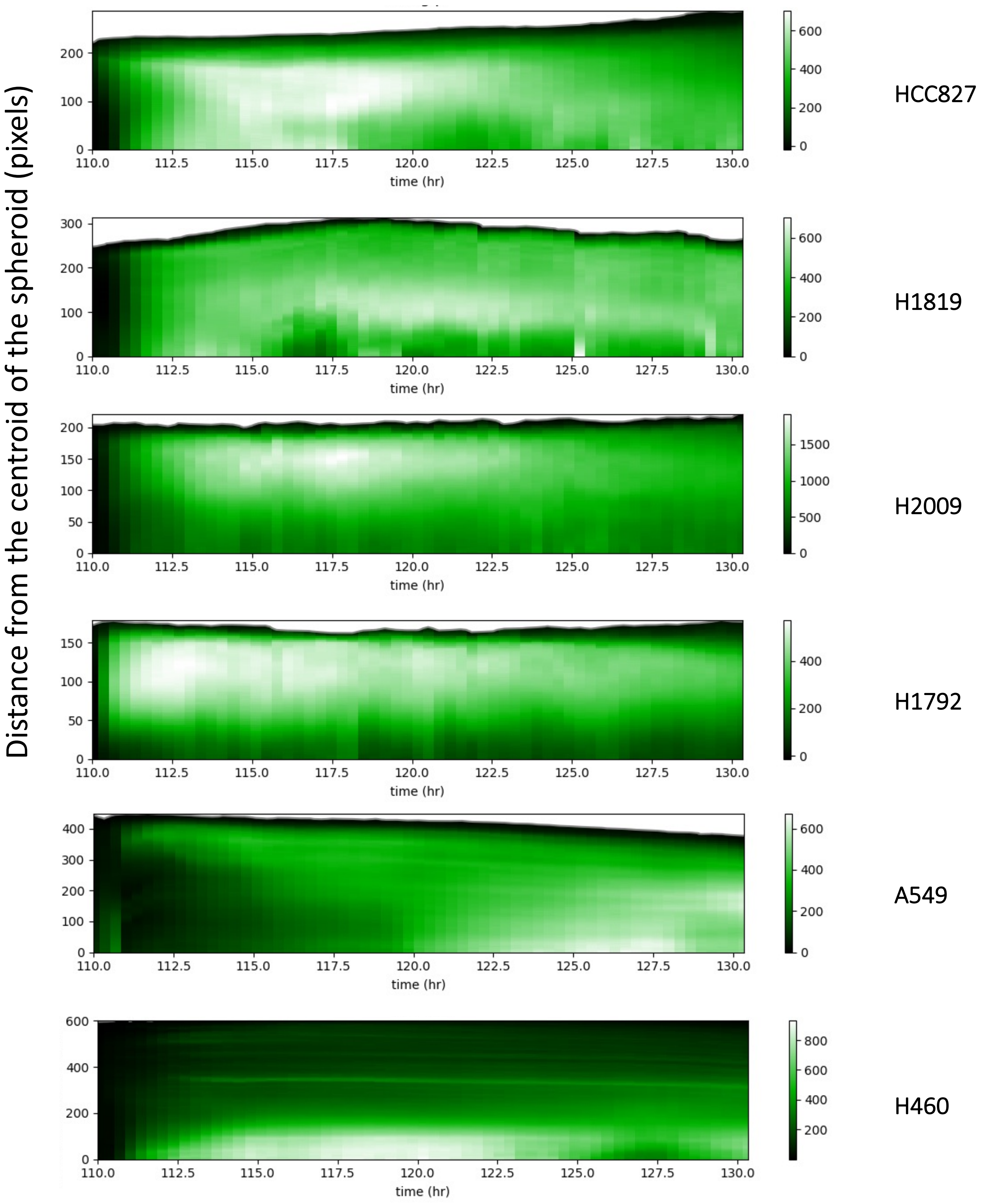

Suppl. Fig. 3: Spatio-temporal dynamics of Salmonella colonizing in NSCLC spheroids

S. typhimurium-spheroid co-cultures were prepared following the protocol in the methods section. The GFP signal from *S. typhimurium* was measured using fluorescence microscopy and the spheroids were imaged with brightfield channel using live cell microscopy. Image analysis was performed to measure the distance of the *S. typhimurium* (GFP) from the center of the spheroid. Representative plots of this value over time are shown here.

Suppl. Fig. 4

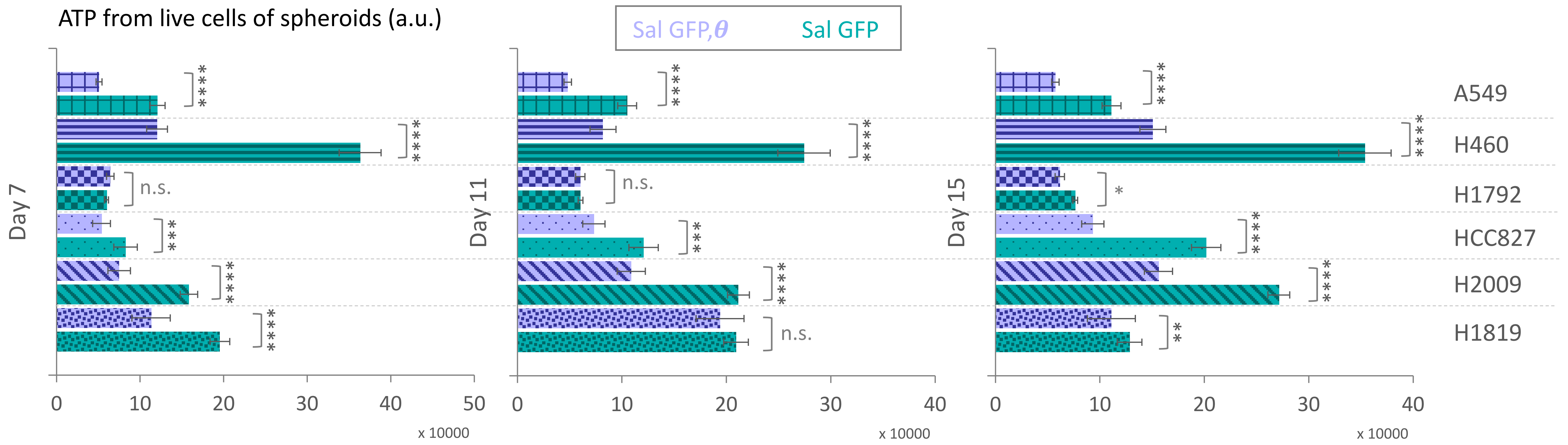

Suppl. Fig. 4: Viability of NSCLC- *S. typhimurium* co-culture spheroids using Cell Titer Glo 3D assay at day 7, 11 and 15 (n=6) after induction of Theta toxin secretion. Significant change (**** = p<0.0001, ***=p<0.001, **=p<0.01, *=p<0.5, n.s.=not significant) was determined by paired, two-tail t-test, and error bars represent standard deviation

Suppl. Fig. 5

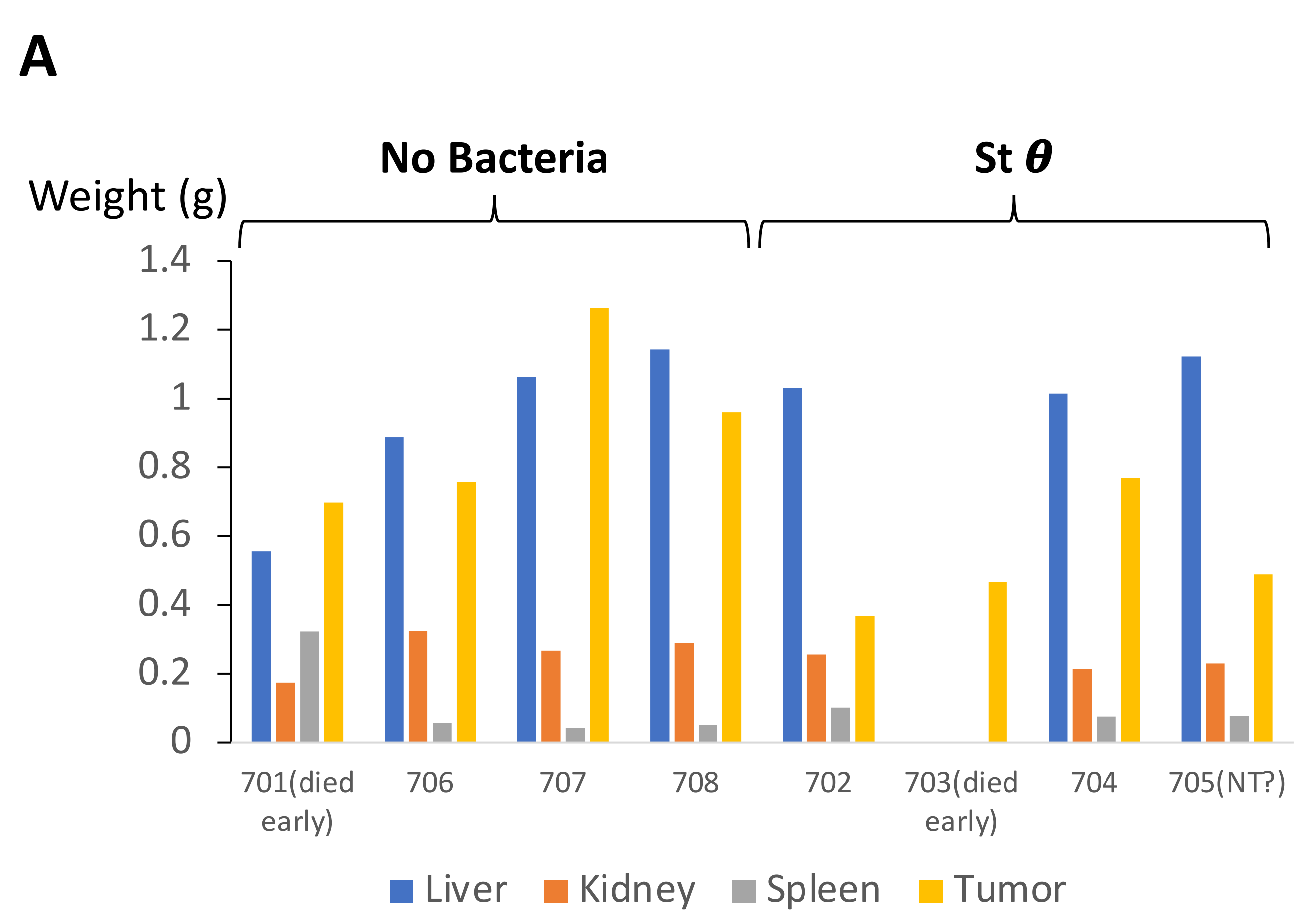

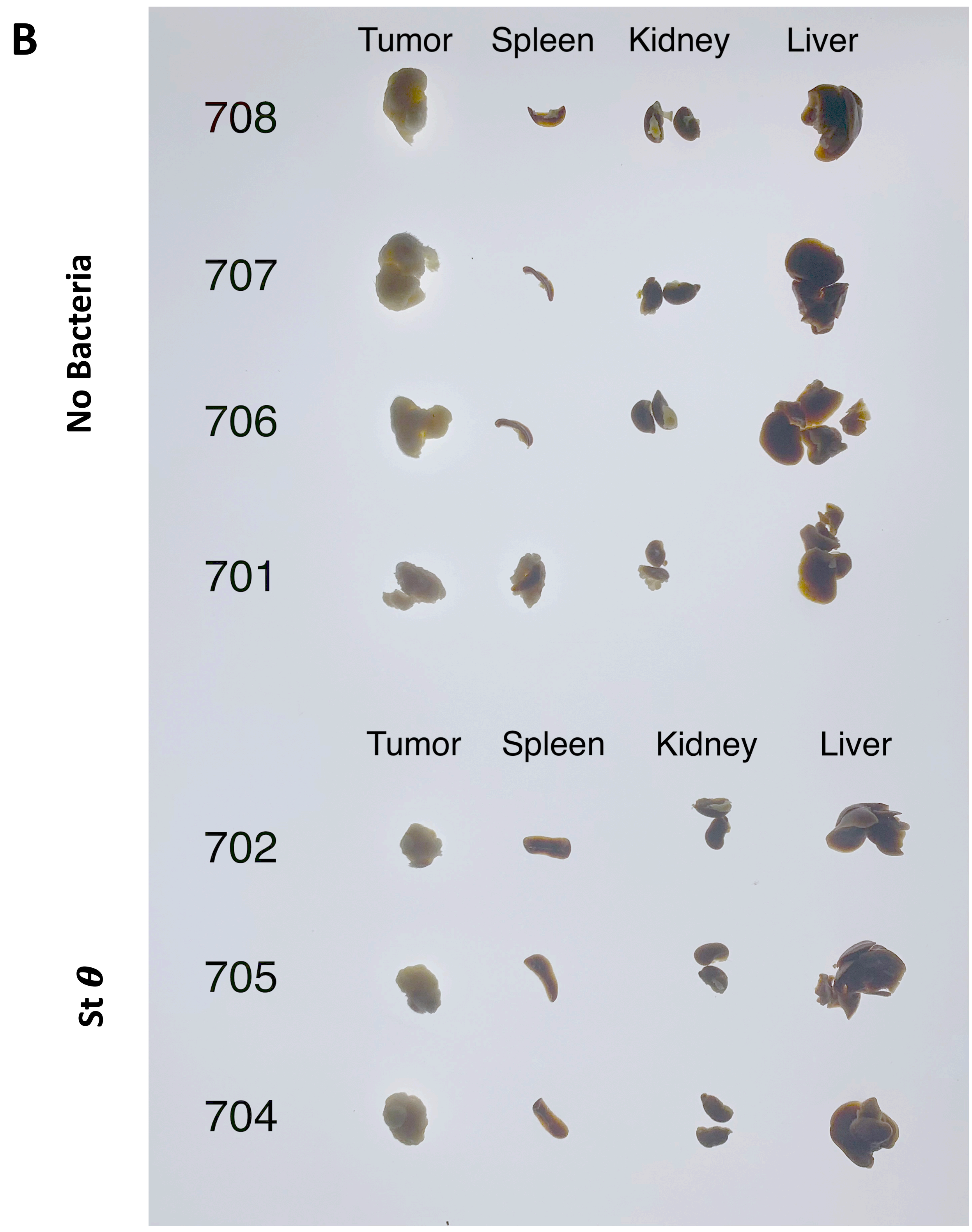

Suppl. Fig. 5: Weight and photo of resected H460 subcutaneous tumors and vital organs after euthanasia
Following euthanasia, tumors and vital organs (liver, spleen, kidney) were collected, gently washed with 70% ethanol, photographed and weighed. For a mouse that died early overnight, vital organs could not be collected. One mouse died during the schedule of treatment.

Suppl. Fig. 6

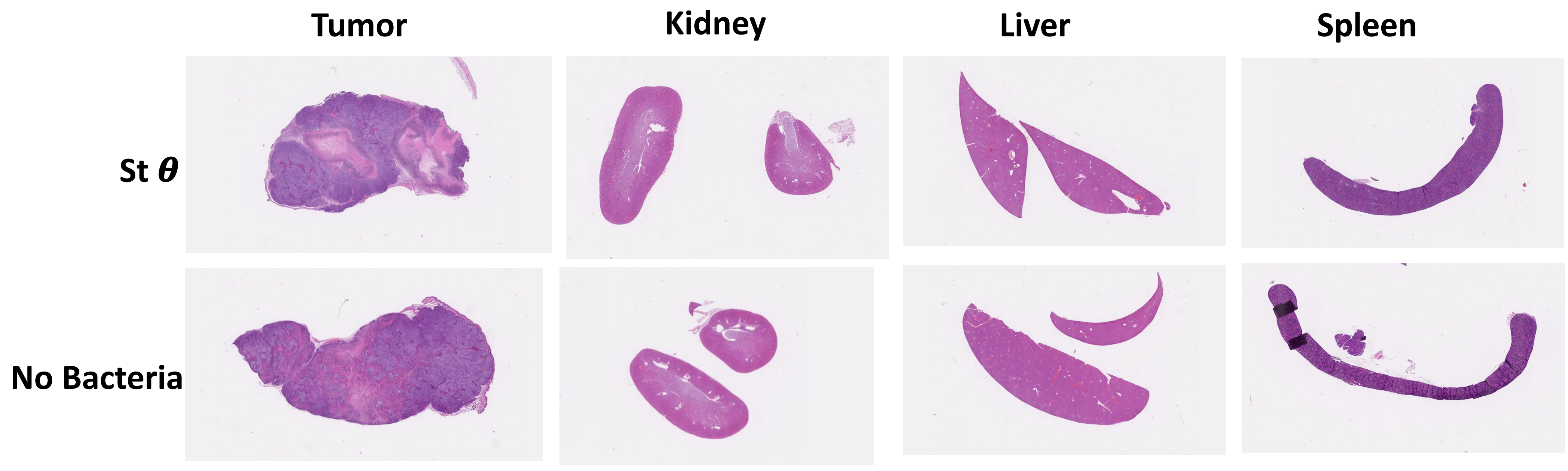

Suppl. Fig. 6: Histology images of tumors and vital organs from NSG mice with H460 subcutaneous tumors and injected with or without bacteria
Representative histology images of formalin-fixed paraffin-embedded and sectioned tumor, liver, kidney and spleen stained with H&E

Suppl. Fig. 7

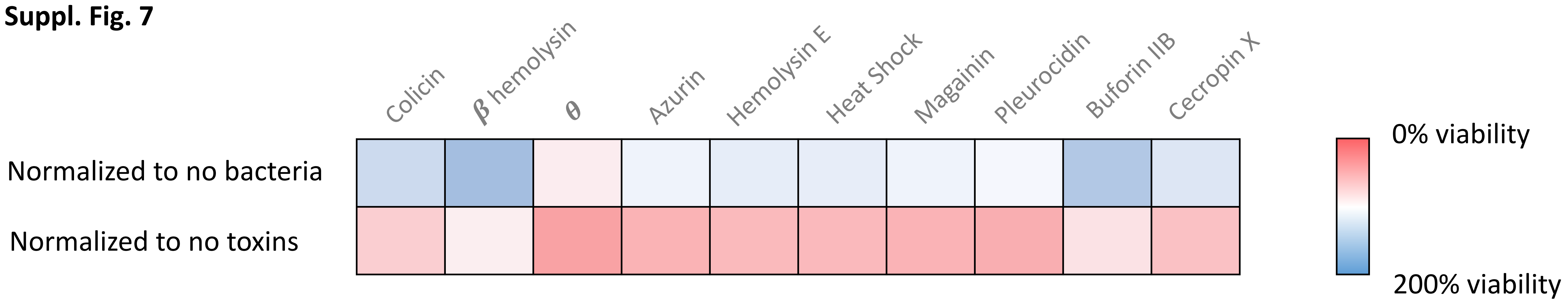

Suppl. Fig. 7: Response of Human Bronchial Epithelial cells (HBECs) lines to bacterially secreted toxins to monolayer assay in batch replicates Fresh lysates of engineered *Salmonella typhimurium* EHL1301 were prepared and were normalized for optical density before adding to the NSCLC monolayer cultures grown in 96-well flat bottom plates. The heatmap represents the median of percent viability (n=8 for plate replicates). Top row represents data normalized to no bacteria control. Bottom row represents data normalized to bacterial lysate without engineered toxins.

Suppl. Fig. 8

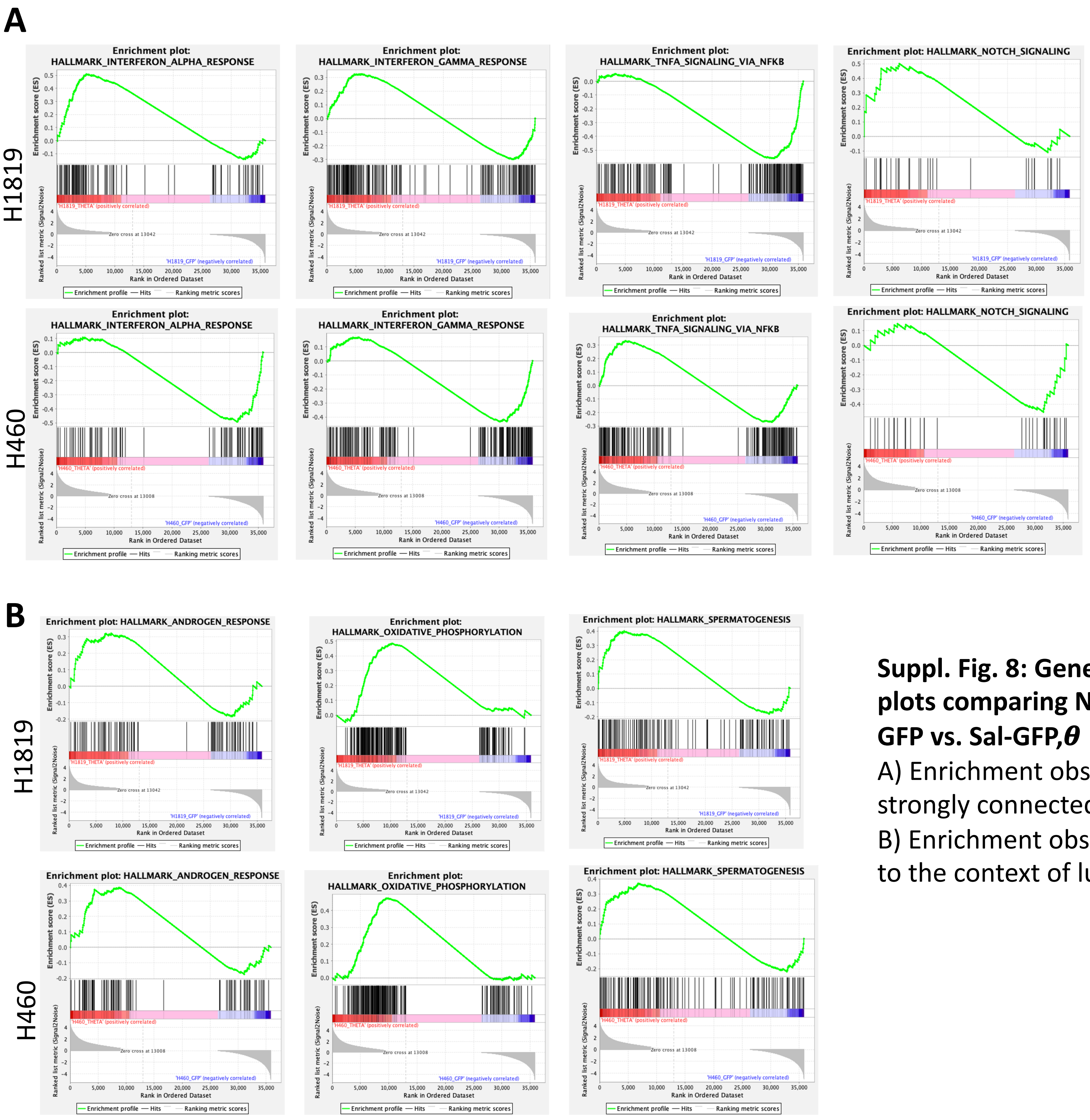

Suppl. Fig. 8: Gene Set Enrichment Analysis (GSEA) plots comparing NSCLC spheroids co-cultured with Sal-GFP vs. Sal-GFP,$\boldsymbol{\theta}$

A) Enrichment observed in one cell line but not in both, strongly connected to lung cancer signaling landscape,

B) Enrichment observed but relatively weakly connected to the context of lung cancer signaling landscape

Suppl. Fig. 9

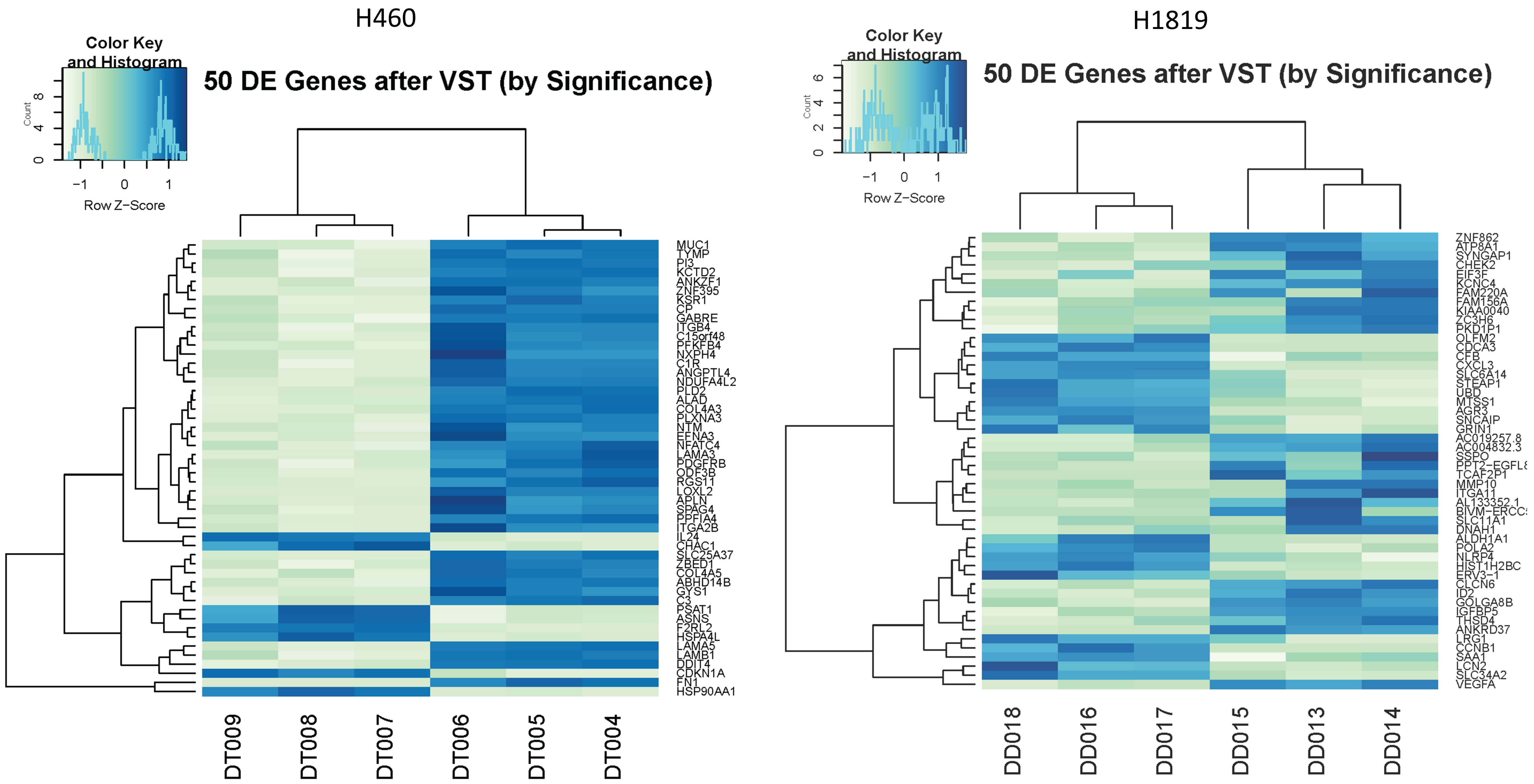

Suppl. Fig. 9: 50 differentially expressed genes across replicates in H1819 and H460 comparing spheroids co-cultured with St vs. St$\boldsymbol{\theta}$, analyzed using DESeq2 (Bioconductor) and Kallisto (Patcher Lab)

Suppl. Fig.10

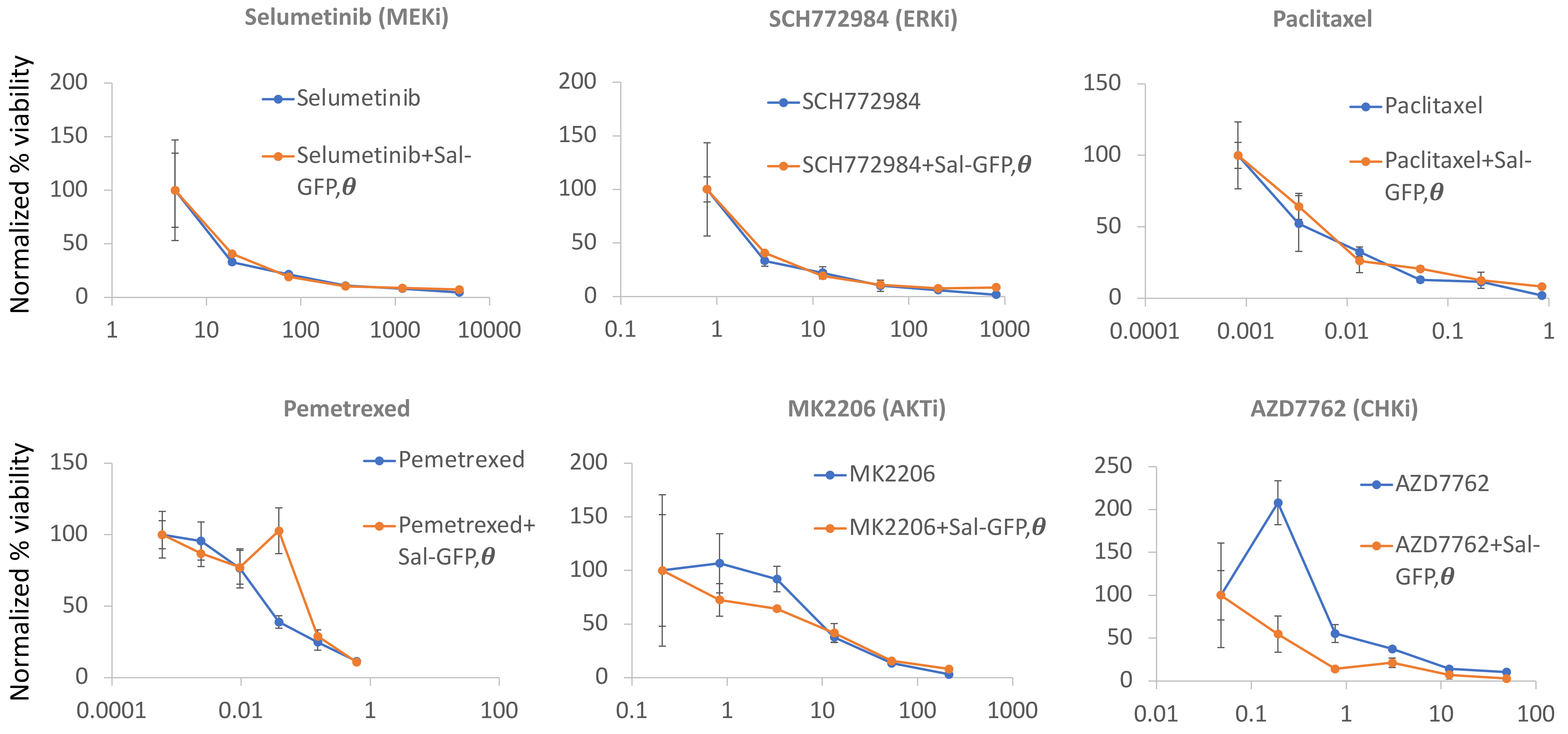

Suppl. Fig. 10: Viability of mouse lung cancer spheroids (with genetically modified TP53 and KRAS), treated with 7 small molecule inhibitors targeting specific signaling identified by GSEA analysis of H460 and H1819, under 2 treatment conditions: 1) Drug only, 2) Drug and bacterially secreted $\boldsymbol{\theta}$ in co-culture After the mouse spheroid and *S. typhimurium* co-cultures were established, AHL was added to induce $\boldsymbol{\theta}$ toxin secretion by the *S. typhimurium* and small molecule inhibitors were added. Error bars represent standard error (n=6). Y-axis represents drug concentrations in μM unit.

Suppl. Fig.11

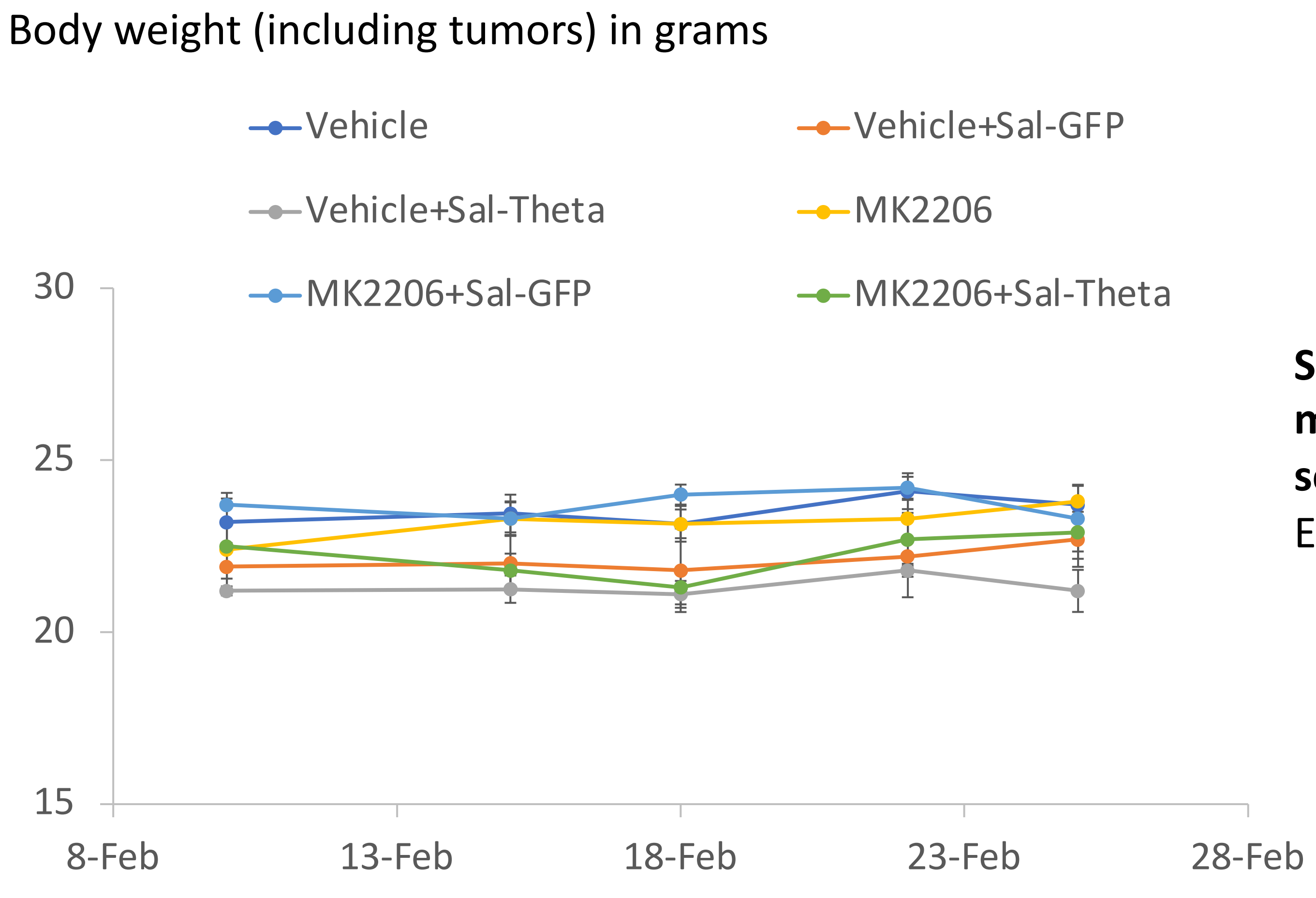

Suppl. Fig. 11: Body weight of mice measured throughout the treatment schedule
Error bars represent standard error (n=3 or 4)
